# Evaluation of Phosphorus Digestibility from Monocalcium and Dicalcium Phosphate Sources and Comparison between Total Tract and Prececal Digestibility Standard Methods in Broilers

**DOI:** 10.3390/ani11123427

**Published:** 2021-12-01

**Authors:** María Cambra-López, Verónica Moset, María del Carmen López, Juan Sebastián Mesa, Laura Carpintero, Andrés Donadeu, Javier Dupuy, Judit Macías-Vidal, Alba Cerisuelo, Pablo Ferrer, Juan José Pascual

**Affiliations:** 1 Institute of Animal Science and Technology, Universitat Politècnica de València, Camino de Vera s/n, 46022 Valencia, Spain; vermoher@hotmail.com (V.M.); malolu@upvnet.upv.es (M.d.C.L.); jupascu@dca.upv.es (J.J.P.); 2 Departamento de I+D+i, Global Feed S.L.U., Grupo Tervalis, Av. Francisco Montenegro s/n, 21001 Huelva, Spain; juan.sebastian@tervalis.com (J.S.M.); laura.carpintero@tervalis.com (L.C.); andres.donadeu@tervalis.com (A.D.); javier.dupuy@tervalis.com (J.D.); judit.macias@tervalis.com (J.M.-V.); 3 Centro de Investigación de Tecnología Animal, Instituto Valenciano de Investigaciones Agrarias, 12400 Segorbe, Spain; cerisuelo_alb@gva.es (A.C.); pfriera@gmail.com (P.F.)

**Keywords:** poultry, mineral nutrition, nutrient availability

## Abstract

**Simple Summary:**

Variations in phosphorous (P) digestibility as a function of methodology, phosphate source, physicochemical characteristics and commercial source were evaluated in broilers. Three methodologies and two phosphates (monocalcium phosphate, MCP, and dicalcium phosphate, DCP) from three different commercial sources were used in two experiments. In the first experiment, MCP and DCP were incorporated into a P-deficient diet at two levels of inclusion and P digestibility was evaluated using three methodologies of the regression method (total excreta, marker in excreta and prececal digestibility). In the second experiment, variations in P digestibility of six phosphate sources (three MCP and three DCP) were evaluated using the total collection method. The P digestibility of MCP ranged from 75.2 to 87.4% and from 80.5 to 86.6% for DCP amongst methodologies (*p* > 0.05). Particle size, surface area, degree of crystallinity and impurities varied amongst commercial sources. The P digestibility of the three tested commercial sources of MCP was 79.6% (MCP1), 70.2% (MCP2) and 65.6% (MCP3); *p* > 0.05. The P digestibility of the three tested commercial sources of DCP was 80.1% (DCP1), 77.4% (DCP2) and 71.4% (DCP3); *p* > 0.05.

**Abstract:**

The objective of this study was to compare the total tract (total excreta and marker) and prececal methodologies to determine phosphorus (P) digestibility and to evaluate its variation as a function of the physicochemical characteristics of the inorganic phosphate used (monocalcium, MCP and dicalcium, DCP) from different commercial sources. A total of 176 1-day-old male broilers were used in two digestibility experiments. In Experiment 1, one MCP and one DCP were incorporated in the basal diet at two levels. In Experiment 2, MCP and DCP from three commercial sources were incorporated to the basal diet at one level. Physicochemical characteristics of inorganic phosphates were examined, as well. Additionally, bone mineralization and growth performance traits were investigated in both trials. The digestibility of MCP ranged from 75.2 to 87.4% and from 80.5 to 86.6% for DCP amongst methodologies, but differences between total tract and preceal methodologies were not statistically significant. Particle size, surface area, degree of crystallinity and impurities varied amongst commercial sources. The P digestibility of the three tested commercial sources of MCP was 79.6% (MCP1), 70.2% (MCP2) and 65.6% (MCP3); *p* > 0.05. The P digestibility of the 3 tested commercial sources of DCP was 80.1% (DCP1), 77.4% (DCP2) and 71.4% (DCP3); *p* > 0.05.

## 1. Introduction

In poultry diets, inorganic phosphorus (P) is added to meet P requirements, as it is an essential mineral for skeletal development and bone mineralization [1]. Inorganic P sources, however, show variability in P digestibility in poultry (ranging from 60% to 91% according to FEDNA [2]). Di-calcium phosphate (DCP), mono-calcium phosphate (MCP) and mono-di-calcium phosphate (MDCP) are the most commonly used forms of inorganic feed phosphates in poultry feed [2].

Matching the nutrient supply precisely with the nutrient requirements of animals is the basis of precision feeding. This is necessary in the interest of safe, high quality and efficient production, while ensuring the lowest possible load on the environment [3]. Therefore, precise knowledge on the P availability of mineral sources is required to adjust diets to animal’s requirements and to obtain the consequent economic and environmental advantages [4].

There are different approaches to evaluate P digestibility (and/or availability) from inorganic phosphate. These approaches can be grouped into three categories: (i) qualitative approaches generally based on bone response characteristics (bioavailability assays), (ii) quantitative approaches based on balance trials (digestibility measurements) and (iii) in vitro assays to determine phosphate solubility [5]. Among these approaches, measuring digestible P through balance digestibility trials is considered the preferred approach [6]. Using this quantitative approach, P digestibility of the test source is calculated by common-intercept multiple regression analyses through regression of total tract (excreta) or ileal digestible P in the diet on added P from the test source [6]. This method requires a basal diet and supplementation with at least two concentrations of a test source. Maintaining dietary P below that which is required is a prerequisite for the linear regression function.

Recently, alternative quantitative approaches to the WPSA protocol [6] have been assayed to evaluate P digestibility from mineral phosphates, plant and animal sources: the direct method [7,8] and the precision-fed chick assay [9]. The direct method requires a semi-purified P-free diet and thus can be considered impractical because semi-purified diets (based on corn starch and sucrose) can alter feed intake. The precision-fed chicken assay is complex as it is based on feeding 6 to 10 g of the test ingredient directly to the animals and collecting ileal digesta approximately 6 h later.

Despite method deviations, most quantitative methods rely on apparent total tract digestibility (ATTD) measurements. The ATTD of P (ATTDP) can be calculated by recording total ingested feed P and total excreted P using the total collection method (ATTDP-tc) or indirectly by using an indigestible marker in the feed (ATTDP-m). Titanium dioxide (TiO_2_) is generally used as an indigestible marker [10]. The ATTDP can also be measured at the terminal ileum level (prececal digestibility of P, pc-DP), because it is believed that the process of P absorption is almost completed in the lower ileum [11]. Moreover, pc-DP is a measurement that stays linear over a wider range of increments in dietary P compared with ATTDP [12]. The ATTDP methods result from and can be influenced by undigested dietary P, endogenous P, P used by hindgut microflora and P excreted via urine [13], while pc-DP does not account for endogenous losses, is unaffected by post-ileal microbial activity and excludes urinary excretion [14].

Previous studies performed at a marginal P level of supply to avoid endogenous excretion have shown that there were no significant differences between ATTDP-tc and pc-DP approaches for a given inorganic phosphate source with the regression technique [12]. An et al. [8] and Munoz et al. [9], however, found pc-DP exhibited higher results compared with ATTDP for meat and bone meal and various phosphates, including MCP and DCP.

The contribution and variability associated with the quantitative method used (ATTDP-tc, ATTDP-m and pc-DP) in the framework of the regression method used to determine the digestible P of mineral phosphate sources has not been addressed exhaustively. The extent to which the methodology used can affect results is still unclear. To our knowledge, no study has been conducted comparing ATTDP-tc, ATTDP-m and pc-DP methodologies. This data would be useful to enable comparison among different studies.

Additionally, P digestibility from mineral P sources depends on factors such as the nature of the product, chemical structure, rate of polymerisation, crystal structure, processing applied before use, and particle size, among others [15]. Differences in digestible P can be attributable not only to phosphate characteristics but also to animal-related factors (the bird’s age, for instance) [12] and diet-related factors (Ca:P ratio or phytate content, for instance) [16]. Information on the effect of intrinsic physicochemical phosphate characteristics such as solubility, particle size, crystallinity and impurities on P digestibility values is particularly scarce. Fulfilling this gap would contribute to improving the understanding on the impact of phosphate-related factors.

To this end, the objective of this study was to compare ATTDP-tc, ATTDP-m and pc-DP methodologies to determine P digestibility calculated using the regression method proposed by WPSA [6] and to evaluate variation in P digestibility as a function of the physicochemical characteristics of the inorganic phosphate used (MCP and DCP) from different commercial sources. Two experiments were conducted. In the first experiment (Experiment 1), one MCP and one DCP were incorporated to a P-deficient diet at two levels of inclusion to evaluate P digestibility using ATTDP-tc, ATTDP-m and pc-DP quantitative methodologies. In the second experiment (Experiment 2), using ATTDP-tc, variations in P digestibility as a function of the phosphate used (MCP and DCP) from three commercial sources was evaluated. Physicochemical characteristics of inorganic phosphates were examined, as well. Additionally, bone mineralization and growth performance traits were investigated in both trials.

## 2. Materials and Methods

All experimental procedures used in this study were approved by Universitat Politècnica de Valencia’s Animal Experimentation Ethics Committee and authorised by the Valencian Conselleria de Agricultura, Medio Ambiente, Cambio Climático y Desarrollo of Spain with the code 2017/VSC/PEA/000166.

### 2.1. Animals

A total of 176 1-day-old male broilers (Ross 308) were used in both experiments distributed in 2 consecutive batches (88 broilers per batch). Each batch had a total duration of 25 days.

### 2.2. Test Products

Six test products (three MCP and three DCP) were used. All products were rock phosphates acquired from different suppliers (Global Feed, Huelva, Spain; Phosphea, France; Yara, Finland and Aliphos, Belgium). In Experiment 1, MCP and DCP (from the same commercial source; MCP1 and DCP1) were tested at 2 levels (0.75 and 1.50 g of added P from the test source/kg). In Experiment 2, MCP and DCP from three commercial sources each (MCP1, MCP2, MCP3, DCP1, DCP2 and DCP3) were compared at one level (1.50 g of added P from the test source/kg). The MCP1 and DCP1 used in Experiments 1 and 2 were acquired from the same supplier but differed in the production batch. Table 1 describes the main physicochemical characteristics of the inorganic phosphates evaluated in this work.

### 2.3. Diets

All animals in both experiments received a commercial pre-experimental starter feed during the first 15 days containing 20.5% crude protein, 3.6% crude fat, 2.6% crude fibre, 6.6% ash, 1.00% calcium (Ca), 0.69% P, 0.15% sodium (Na), 0.5% methionine and 1.14% lysine. On day 15, all animals were fed the experimental diets until day 25.

Experimental feeds were manufactured based on a common basal diet formulated to meet nutritional requirements established by Santomá and Mateos [17] for broilers that age, except for total P and Ca. The basal diet was manufactured in mash form, including TiO_2_ as the indigestible marker (0.5%). The basal diet was free from phytase, microorganisms, essential oils or medication. A single vitamin and mineral premix was added which contained 1440 IU per kg of feed of vitamin D3.

Table 2 shows the ingredients of the basal diet in both experiments. The different experimental diets were formulated based on the analyzed P and Ca contents of the basal diet and their content in each phosphate test product (Table 1) to achieve the desired amount of total P and a constant total Ca:total P relation of 1.34. Therefore, limestone and each phosphate were added in different proportions to the basal diet. Supplements were made at the expenses of diatomaceous earth (P-free ingredient) from the basal diet. At the highest inclusion level (1.5 g of added P from the test source/kg), the total P was 3.8 g total P/kg, which is below published requirements for broilers [17].

In Experiment 1, MCP1 and DCP1 were incorporated in the basal diet at two levels (Level 1 = 0.75 and Level 2 = 1.50 g of added P from the test source/kg). This resulted in five different experimental diets: BS, basal diet without added P; MCP1 Level 1, BS + 0.75 g of added P from MCP1/kg; MCP1 Level 2, BS + 1.50 g of added P from MCP1/kg; DCP1 Level 1, BS + 0.75 g of added P from DCP1/kg; and DCP1 Level 2, BS + 1.50 g of added P from DCP1/kg.

In Experiment 2, MCP and DCP from 3 commercial sources (MCP1, MCP2, MCP3, DCP1, DCP2 and DCP3) were incorporated to the basal diet at one level (Level 2 = 1.50 g of added P from the test source/kg). This resulted in seven different experimental diets: BS, basal diet without added P; MCP1 Level 2, BS + 1.50 g of added P from MCP1/kg; MCP2 Level 2, BS + 1.50 g of added P from MCP2/kg; MCP3 Level 2, BS + 1.50 g of added P from MCP3/kg; DCP1 Level 2, BS + 1.50 g of added P from DCP1/kg; DCP2 Level 2, BS + 1.50 g of added P from DCP2/kg; and DCP3 Level 2, BS + 1.50 g of added P from DCP3/kg. Table 3 shows the nutrient composition of each experimental diet.

Water and diets were offered ad libitum during the whole study. In addition, hydrochloric acid was used to acidify drinking water to a pH between 6.5 and 7.

### 2.4. Experimental Procedure

Over the experimental period, ventilation rates and room temperature were adapted to the age of the animals. The temperature ranged from 24 to 32 °C. The light program started with 24 h of light and 0 h of darkness; the ratio of light:darkness was gradually adapted during the first six days until it reached 18:6, which was maintained until the end of the experiment.

During the pre-experimental period (first 15 days), all animals were allocated in a single pen with metallic feeder troughs and nipple drinkers. The floor was covered with 10 cm of wood shavings.

On day 15 of the study, all animals were weighed and distributed in pairs in metabolic cages (56 × 54 cm^2^, eight cages per treatment) for 10 days. During this period, animals were fed the experimental diets. The two animals housed in the same cage were selected according to similar body weights (BW) to ensure homogeneous feed consumption. The average BW amongst treatments ± standard deviation on day 15 was 487.5 ± 0.96 g (Experiment 1) and 487.8 ± 1.79 g (Experiment 2). Each cage contained a nipple drinker and a feeder trough. In total, 44 metabolic cages per batch were used. There were eight replicates/treatment. Both experiments were performed at the same time in both batches.

The duration of the cage phase was subdivided into two periods: a 6-day adaptation period (adaptation to cages and diets) and a 4-day digestibility trial. During the digestibility trial, feed intake and excreta output were controlled daily. To determine ATTP-tc and ATTP-m, excreta were collected daily during the last four days from trays underneath each cage. Daily samples were pooled per cage and stored at −20 °C until analyses.

At the end of the experiment, animals were weighed and slaughtered by electric stunning and exsanguination. To determine pc-DP, digesta content was collected from the ileum. At slaughter, the abdominal cavity of each bird was opened and the ileum of the birds was immediately dissected. The section between Meckel’s diverticulum and 2 cm prior to the ileo-caeco-colonic-junction was excised. The terminal two-thirds of the section were used for digesta sampling as suggested by Rodehutscord et al. [16]. Digesta content was flushed out with distilled water for all birds and pooled per cage (two animals per cage). Digesta samples were immediately frozen at −20 °C until analyses.

Feed consumed was measured per cage. The average daily gain (ADG) and feed conversion ratio (FCR) were calculated on a cage basis from ADG and average daily feed intake (ADFI) data. Additionally, the left tibia of all broilers was removed and, after removing all the soft tissues, frozen at −20 °C until analyses. The two left tibias per metabolic cage were pooled and analyzed.

### 2.5. Analytical Methods

Phosphates were analyzed according to Commission Regulation (EC) No. 152/2009, which lays down the methods of sampling and analysis for the official control of feed, and Regulation (EC) No 2003/2003, which relates to fertilizers.

Feed samples were dried at 105 °C for 24 h, and excreta samples were dried at 80 °C for 48 h, after which they were ground (0.5-mm sieve) and stabilized at room temperature. Digesta samples were freeze-dried and ground, as well.

Each experimental diet, excreta and digesta contents were analyzed for dry matter (DM) and P. In addition, diets were analyzed for Ca. Analyzed P and Ca concentrations in feed confirmed intended values.

The DM content in feed, excreta and digesta was analyzed according to method 934.01 of AOAC [18]. Mineral (Ca and P) content was analyzed by inductively coupled plasma atomic emission spectrometry (ICP-OES) (model Varian 720-ES, Varian Inc., Palo Alto, CA, USA). In brief, a dried and ground subsample of 3–5 g of each feed, excreta and digesta sample was ashed at 550 °C for 3.5 h in a muffle furnace. Samples were cooled, and 4 mL concentrated HCl (37%), 1 mL HNO_3_ and 1 mL of Ytrium solution (100 mg/L) was added to a 0.1 g-ashed sample. Samples were then filtered through a nylon 0.45 μm filter, and the filtered solution was analyzed in the ICP-OES. Marker concentration (TiO_2_) was analyzed in feeds, excreta and digesta according to the methodology described in Short et al. [19].

For the determination of ash, Ca and P in tibias, bones were dried at 110 °C for 12 h, defatted with an ether solution for 48 h and dried at 110 °C for 12 h, as described in Català-Gregori [20]. Bones were then weighed and ashed at 550 °C for 12 h in a muffle furnace. The ash content was expressed as a percentage of dry fat-free bone weight. Mineral (Ca and P) content in tibia bones was then analyzed using ICP-OES by adding 0.05 g ashed sample to the acid solution instead of 0.1 g, as described above.

### 2.6. Phosphorus Digestibility Calculations

The digestibility of DM and P from experimental diets in Experiment 1 was calculated using three approaches: ATTD-tc, ATTD-m and pc-D. The digestibility of DM and P from experimental diets in Experiment 2 was calculated using ATTD-tc.

The ATTD-tc of experimental diets was calculated following Equation (1):

ATTD-tc (%) = ([(Feed intake × Nutrient_diet_) − (Excreta output × Nutrient_excreta_)])/(Feed intake × Nutrient_diet_) × 100 (1)

where Nutrient_diet_ and Nutrient_excreta_ were the analyzed concentrations of DM or P in the diet (g/kg) and excreta (g/kg), respectively, in a dry basis.

The ATTD-m of experimental diets was calculated following Equation (2):

ATTD-m (%) = 100 − [100 × (Ti_diet_ × Nutrient_excreta_)/(Ti_excreta_ × Nutrient_diet_)] (2)

where Ti_diet_ and Ti_excreta_ were the analyzed concentrations of TiO_2_ in the diet (g/kg) and in the excreta (g/kg), respectively, while Nutrient_diet_ and Nutrient_excreta_ were the analyzed concentrations of DM or P in the diet (g/kg) and excreta (g/kg), respectively, in a dry basis.

The pc-D of experimental diets was calculated following Equation (3):

pc-D (%) = 100 − [100 × (Ti_diet_ × Nutrient_digesta_)/(Ti_digesta_ × Nutrient_diet_)] (3)

where Ti_diet_, Ti_digesta_ and Nutrient_diet_ and Nutrient_digesta_ were the analyzed concentrations of DM and P in the diet (g/kg) and digesta content (g/kg), respectively, in a dry basis.

To determine the P digestibility of each phosphate, we followed the WPSA [6] protocol, where digestible P (expressed in g/kg of diet) was plotted against the P added from the test product (g/kg of diet) in a in a common-intercept linear regression analysis. The slope of the regression line, multiplied by 100, gave the percentage digestibility of P from the supplemented source. Digestible P content (g/kg) in the diet was calculated by multiplying the ATTDP-tc, ATTDP-m and pc-D of P (%) by P content (g/kg) in the diet, divided by 100.

### 2.7. Field Emission Scanning Electron Microscopy for Sample Surface and X-ray Elemental Microanalysis

To investigate the physical characteristics and elemental analyses (impurities) of phosphate sources used in Experiment 2, samples were analyzed using a field emission scanning electron microscope (FESEM) (ZEISS AURIGA Compact model). Scanning electron data were used to obtain information from the sample surface (topography, surface area and crystallinity).

A secondary electron in-lens detector was used to provide high-resolution images at low accelerating potentials (<5 kV), minimizing the charging effect on non-conductive specimens and the sample damage of electron beam-sensitive samples.

Furthermore, microanalysis by an X-ray dispersive energy detector (EDS) (Oxford Instruments) was used to provide qualitative and quantitative elemental analytical information about selected areas on the surface of the sample.

A sample of each phosphate was mounted on a carbon stub, coated with carbon and used in FESEM analyses. Photomicrographs were taken in each sample at 50× and 3000× and 10,000× magnification. At least six sites of interest were sampled in each phosphate.

### 2.8. Statistical Analysis

Data were statistically analyzed using the SAS Software 9.3 statistical program. The cage served as the experimental unit for all statistical analysis (*n* = 8 per treatment). For growth performance and digestibility, the average of the two animals per cage was used. For bone mineralization parameters, the two left tibias per metabolic cage were pooled and analyzed together. The basic statistical model employed was ANOVA. Significant differences were declared at *p* ≤ 0.05. Data from Experiments 1 and 2 were analyzed separately.

In Experiment 1, the GLM procedure of SAS was used to analyze performance, bone mineralization and digestibility parameters. The experimental treatment, including phosphate type (MCP or DCP), phosphate level (0, 0.75 and 1.5 g/kg) and batch, were considered as fixed effects in all parameters evaluated. Initial body weight was used as a covariable in bird performance data. The effect of the method used to determine P digestibility of the diet and phosphate (ATTD-tc, ATTD-m and pc-D) was evaluated, as well. Regression equations were calculated from each data set using the REG procedure of SAS restricting the intercept to the P digestibility (g/kg) average of the basal diet. The slopes derived from each method for MCP and DCP were compared using the SLOPE DIFFERENCE tool of the REG procedure of SAS.

In Experiment 2, the GLM procedure of SAS was used to analyze performance, bone mineralization and digestibility parameters. The experimental treatment, including commercial phosphate source (MCP1 to 3 and DCP1 to 3) and batch, were considered as fixed effects in all parameters evaluated. Initial body weight was used as a covariable in bird performance data. Data were analyzed in the same way as in Experiment 1, but were analyzed independently for MCP and DCP.

Elemental X-ray analyses were analyzed using the GLM procedure of SAS using the experimental treatment as a fixed effect.

## 3. Results

### 3.1. Experiment 1

Animal health was good and no mortality was observed during the experiment. Table 4 shows animal performance traits in Experiment 1 from 15 to 25 days of age (during the whole cage phase including the adaptation and digestibility periods) when animals were fed the experimental treatments. The average BW amongst treatments at the start of the adaptation period (day 15) was 488 g, and the final average BW at the end of the digestibility period (day 25) was 812 g. Average daily feed intake ranged from 50 to 58 g/day; ADG ranged from 29 to 37 g/day and FCR ranged from 1.6 to 1.8. Dietary treatments had no influence (*p* > 0.05) on BW, ADFI, ADG and FCR between 15 and 25 days in Experiment 1.

Phosphate levels influenced mineralization traits (Table 4). The basal diet showed the lowest (*p* < 0.05) ash % and P and Ca (in mg). There was a clear response in tibia bones when administered graded levels of phosphates. Generally, MCP and DCP Level 1 (0.75 g of added P from the test source) and Level 2 (1.50 g of added P from the test source) showed higher mineralization traits (tibia ash and mineral content) compared with basal diet (*p* < 0.05), except for tibia weight, where only Level 2 treatments showed statistically significant differences with the basal diet. Dietary treatments had no influence (*p* > 0.05) on % of P and Ca in tibia ash in Experiment 1.

Diet DM and P digestibility coefficients using the ATTD-tc, ATTD-m and pc-D methods are presented in Table 5. Dry matter digestibility coefficients ranged from 78 to 81% and were similar amongst treatments and methods. There were only slight significant differences in DM digestibility coefficients of the diet when using ATTD-tc and ATTD-m amongst treatments. As regards P digestibility, coefficients ranged from 46 to 63%. The level of P in the dietary treatments influenced P digestibility coefficients. The basal diet showed the lowest ATTD-tc, ATTD-m and pc-D values for P. The difference between the basal diet and diets with graded levels of dietary P was significant (*p* < 0.05) with MCP and DCP at Level 1 and Level 2 when using the ATTD-tc and pc-D methods, but was only significantly different when using ATTD-m at Level 2.

Table 6 shows linear regression equations and P digestibility coefficients (derived from the slopes) as a function of the phosphate source and the digestibility method used. P digestibility of MCP1 was 83.5% (ATTD-tc method), 75.2% (ATTD-m method) and 87.4% (pc-D method). P digestibility of DCP1 was 80.8% (ATTD-tc method), 80.5% (ATTD-m method) and 86.6% (pc-D method). Differences amongst method calculations were not statistically significant (*p >* 0.05) (Table 6). Therefore, in the linear models used to estimate P digestibility, the method used did not have a significant effect on the slope for MCP and DCP. All method calculations showed comparable standard errors (ranging from 0.05 to 0.08) and r^2^ (ranging from 0.86 to 0.93).

### 3.2. Experiment 2

Animal health was good and no mortality was observed during the experiment. Table 7 shows animal performance traits in Experiment 2 from 15 to 25 days of age (during the whole cage phase including adaptation and digestibility periods) when animals were fed experimental treatments. Average BW amongst treatments at the start of the adaptation period (day 15) was 488 g, and final average BW at the end of the digestibility period (day 25) was 826 g. Average daily feed intake ranged from 53 to 60 g/day; ADG ranged from 32 to 38 g/day and FCR ranged from 1.6 to 1.8. Dietary treatments had no influence (*p >* 0.05) on BW, ADFI, ADG and FCR between 15 and 25 days in Experiment 2.

Phosphate source did not influence mineralization traits (Table 7). Therefore, there was a similar response in tibia bones when administered MCP and DCP at Level 2, independent form the origin (commercial source).

Dietary DM and P digestibility coefficients are presented in Table 8. Dry matter digestibility coefficients ranged from 78 to 80% and were similar (*p >* 0.05) amongst sources. There were no statistically significant differences in P digestibility coefficients, which ranged from 54 to 60%, amongst treatments.

Table 9 shows linear regression equations and P digestibility coefficients (derived from the slopes) as a function of mineral commercial source using the ATTD-tc method. The P digestibility of MCP was 79.6% (MCP1), 70.2% (MCP2) and 65.6% (MCP3). The P digestibility of DCP was 80.1% (DCP1), 77.4% (DCP2) and 71.4% (DCP3). These differences were not statistically significant (*p >* 0.05).

**Figure 1:**
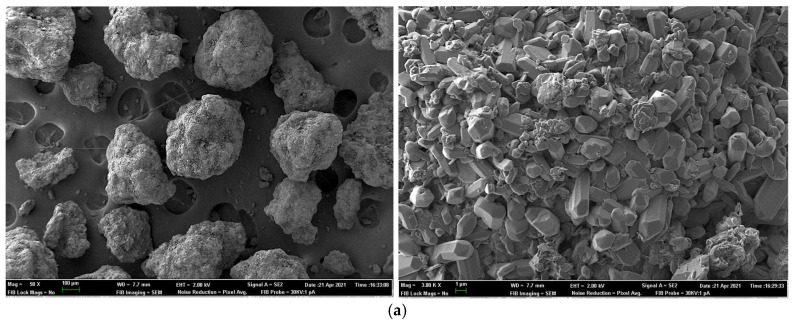

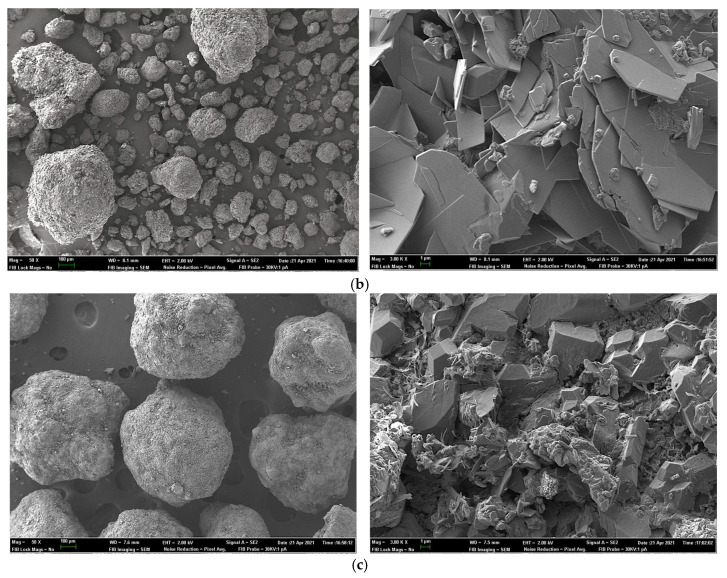
Scanning electron photomicrographs of monocalcium phosphate (MCP) sources: (**a**). MCP1, (**b**). MCP2 and (**c**). MCP3 products at 50× and 3000× magnification showing particle size, surface area and degree of crystallinity of samples.

**Figure 2:**
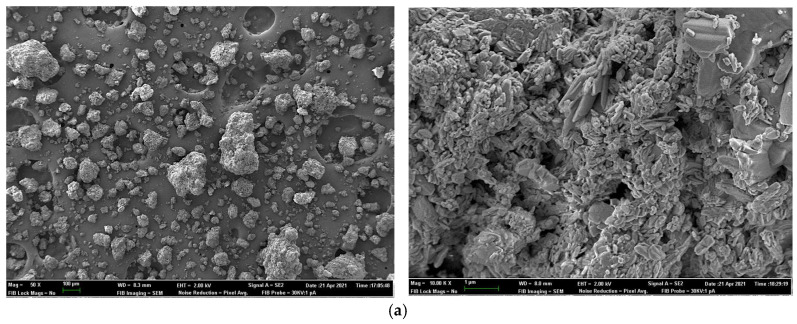

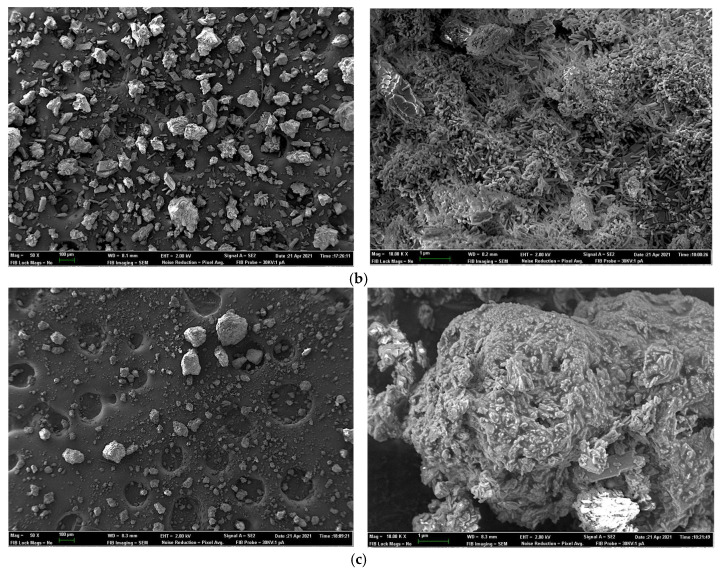
Scanning electron photomicrographs of dicalcium phosphate (DCP) sources showing (**a**). DCP1; (**b**). DCP2 and (**c**). DCP3 products at 50× and 10,000× magnification showing particle size, surface area and degree of crystallinity of samples.

Figure 1 and Figure 2 show the SEM photomicrographs and X-ray analyses of test phosphates. Observations showed particle size, surface area, degree of crystallinity and elemental composition varied amongst sources. Generally, MCP showed larger particles than DCP. MCP1 exhibited a higher surface area and degree of crystallinity than the rest of the MCP sources. DCP1 and DCP2 showed a higher surface area and degree of crystallinity than DCP3.

The X-ray elemental analyses shown in Table 10 and Table 11 revealed the presence of certain impurities in all sources. For MCP (Table 10), MCP1 showed the highest (*p* < 0.05) Na and the lowest (*p* < 0.05) Mg; MCP2 showed the highest (*p* < 0.05) Al, K and Fe; and MCP3 showed the highest (*p* < 0.05) Si contents. For DCP, DCP1 showed the highest (*p* < 0.05) Mg, Al and K; DCP2 showed the highest (*p* < 0.05) Cl and the lowest (*p* < 0.05) Mg; and DCP3 showed mostly intermediate contents. Differences amongst the rest of elements were variable.

## 4. Discussion

This study addressed two independent but directly related objectives to evaluate P digestibility from MCP and DCP phosphates using a single approach: the regression method recommended in WPSA [6]. In Experiment 1, we compared different methodologies to calculate digestible P from phosphate sources, whereas in Experiment 2, we investigated how variations in commercial phosphate source could influence P digestibility. The physical characteristics and elemental analyses (impurities) of phosphate sources were investigated, as well. Additionally, the effect of phosphate type and source on bird’s bone mineralization and growth performance traits was investigated.

In Experiments 1 and 2, dietary treatments had no influence (*p >* 0.05) on bird performance traits. Generally, growth performance variables are not adequate end-point criteria for P availability evaluations [5,13,21]. Differences in growth performance parameters can only be noted when there are large differences in bioavailability or mineral supply [22]. Moreover, the impact on growth performance is related to the administration period of experimental diets containing test products. In our study and in Trairatapiwan et al. [14], short administration periods were used (normally between 5 to 10 days), as these periods are common in assays where digestible P is the target. This length of time is probably not enough to detect differences in performance traits.

In the review conducted in Shastak and Rodehutscord [5], bone criteria (bone ash, primarily) rather than animal performance traits or blood parameters were identified as the most sensitive, suitable and validated measurement criteria for estimating P availability from different P sources in broilers. In Shastak et al. [23], a very strong effect of P level on tibia ash, tibia P and tibia Ca was found. These authors stated that bone-mineralization criterion are more sensitive indicators of the P status of the birds than growth performance parameters. This fact was also stated by Ravindran et al. [24], because the bone is the main storage organ for P, containing 85% of the body’s total P.

In our study, although animals were fed experimental diets for a short period of time (10 days), bone mineralization traits were influenced by phosphate level (Experiment 1) but not by phosphate type or source (Experiments 1 and 2). Hemme et al. [25] and Hamdi et al. [26] concluded that despite the different physical structure and chemical properties amongst MCP and DCP, no evidence was observed regarding differences in tibial parameters. However, Lamp et al. [27] found significantly higher tibia weight and ashes in MCP compared with DCP in 21-day-old broilers.

Our data from Experiment 1 showed that the digestibility of MCP ranged from 75.2 to 87.4% and the digestibility of DCP ranged from 80.5 to 86.6% among methodologies. In general, results obtained in this work are slightly higher than those reported in the literature for broilers. For instance, these values are slightly higher than those described in Spanish nutrient feed tables [2], where the proposed value for P digestibility in broilers is 83% for MCP and 79% for DCP, and also slightly higher than those reported by Trairatapiwan et al. [14]. Shastak et al. [12] indicated that the values of P digestibility of DCP ranged from 25 to 30% when two different methodologies (ATTDP-m and pc-PD) were used with two different aged groups of birds, whereas in Bikker et al. [28], the pc-DP digestibility for MCP was 78.3% and 59.0% for DCP. In another study, van Harn et al. [29] presented a pc-DP digestibility equal to 88.5% for MCP and equal to 82.4% for DCP in broilers.

As regards comparison between methodologies (Experiment 1), our data showed that when an indigestible marker was involved in ATTDP-m, comparable P digestibility values were obtained with ATTDP-tc. The full recovery of markers can be limiting when using such methodologies [30,31,32]. This was not the case in our study, where the average recovery of TiO_2_ in excreta in all pooled samples from Experiment 1 was 104 ± 11.0% (data not shown). These results indicate that in the conditions of our study, both ATTDP-tc and ATTDP-m can be used indistinctly.

Theoretical differences between ileal P digestibility and total tract digestibility of P could occur either by P excretion with urine or postileal absorption and secretion of P [12]. Furthermore, differences between the ATTDP and pc-DP methods could be related with compensation mechanisms when broilers are fed P-deficient diets. An et al. [8] outlined that, when Ca and P intake is low, the mobilization of Ca and P from bone can be promoted to maintain homeostasis, and thus, a portion of mobilized P can be found in the excreta, resulting in low ATTDP values. This would lead to the underestimation of ATTDP (either tc or m method) calculated via excreta compared with pc-DP. Nevertheless, Ca levels tend to be low, because excess Ca is known to negatively affect P solubility due to Ca-P complexes in the intestinal lumen [33].

Munoz et al. [9] presented differences amongst ileal and total tract methods, showing generally lower P digestibility coefficients when using ATTDP compared with pc-DP in diets. These authors pointed out two explanations for those results: the first involving low-Ca unbalanced diets that can limit P absorption, and the second related to the fact that total tract excreta *p* values are “apparent” values and are not corrected for endogenous P losses, and, thus, are not corrected for urinary P loss. According to Mutucumarana and Ravindran [34], endogenous P losses are composed of bile secretions (containing phospholipids) to a wide extent, as well as enzyme secretions, sloughed epithelial enterocyte cells and gut microorganisms.

Linear regression models using different methods showed comparable errors and r2. High variabilities in pc-DP have been previously found in literature, as in [17,35]. This could be attributable to the inclusion of marker analysis and lab determination, which comprises an additional source of error. In our study, however, standard errors in pc-DP were only slightly higher compared with ATTDP methods, and ATTDP-tc and ATTDP-m showed very similar results, thus pointing to a high degree of accuracy in marker determination both in feed and excreta.

As regards phosphate type and commercial source (Experiment 2), Bikker et al. [28] observed that commercial MCP sources significantly affected P digestibility. In Experiment 2, three different commercial sources of each mineral source were compared, and there were no significant differences on mineral digestibility or bone mineralization parameters among commercial sources. Carpintero et al. [36], however, using the same MCP phosphates, reported significant differences between MCP3 and the rest of commercial sources tested.

Lima et al. [37] indicated that particle size could influence the P digestibility of inorganic phosphates. Phosphorus from large particle sizes (containing coarse particles) were shown to be more biologically available to broilers, probably due to longer retention times in the gizzard under more acidic conditions that may solubilize P more completely [38]. The chemical formula of phosphate could affect the chemical form in which P is present and could also interfere with P digestibility. The percentage of MCP and DCP in each phosphate type could vary considerably. Higher proportions of purified-grade MCP are related to improved P availability, as in [38,39]. Commercial DCP products are generally a mixture of varying amounts of DCP and MCP, phosphoric acid, calcium carbonate and impurities [37]. The final mixture highly depends on the origin of the rock phosphates and acids used and on the industrial production process employed. Moreover, van Harn et al. [29] established a positive relationship between the spatial structure of phosphates using SEM images (surface area of the test product) with pc-DP. The higher the surface area and the higher the degree of crystallinity, the higher the digestibility coefficient. A larger area might enhance solubilization of P in the small intestinal tract and thus improve intestinal absorption of P [29]. These results could also relate well with P solubility data.

In summary, the direct comparison of P digestibility coefficients obtained from different studies and calculated from different experimental criteria is complex [40]. Therefore, it is of utmost importance to reduce bias related to the experimental methodology (regression, direct and precision-fed chickens) but also within the same experimental methodology (i.e., regression methods). Particle size and intrinsic physical characteristics of phosphates also need to be taken into account. This would improve comparisons and clarify whether differences obtained amongst studies are attributable to phosphate-related factors or extra-phosphate-related ones.

## 5. Conclusions

The P digestibility of MCP in our study ranged from 75.2 to 87.4%; and the P digestibility of DCP ranged from 80.5 to 86.6% amongst ATT-tc, ATTD-m and pc-D methodologies. Differences between total tract and preceal methodologies were not statistically significant. Particle size, surface area, degree of crystallinity and impurities varied amongst sources. The P digestibility of the three tested commercial sources of MCP was 79.6% (MCP1), 70.2% (MCP2) and 65.6% (MCP3); *p >* 0.05. The P digestibility of the three tested commercial sources of DCP was 80.1% (DCP1), 77.4% (DCP2) and 71.4% (DCP3); *p >* 0.05.

## Figures and Tables

**Table 1:** Analyzed physicochemical characteristics of monocalcium (MCP) and dicalcium (DCP) phosphates used.

	Experiment 1		Experiment 2					
Inorganic Phosphates	MCP1	DCP1	MCP1	MCP2	MCP3	DCP1	DCP2	DCP3
Dry matter (g/kg)	990	985	901	965	983	983	982	955
Phosphorus (P) (g/kg)	230	183	229	224	212	181	196	183
Calcium (g/kg)	167	244	168	160	183	248	256	287
P solubility in water (%)	87.3	58.1	88.6	79.0	80.2	50.0	3.2	0.1
Particle size (%)								
>2.50 mm	0.00	0.00	0.00	0.00	0.00	0.00	0.00	0.00
>2.00 mm	0.20	0.00	0.50	0.1	2.70	0.00	0.00	0.00
>1.80 mm	1.50	0.00	1.10	0.20	5.20	0.00	0.00	0.30
>1.60 mm	25.0	0.50	18.0	0.30	9.20	0.70	0.20	0.30
>1.25 mm	37.0	1.30	32.0	5.20	23.1	2.10	0.50	0.70
>0.40 mm	97.0	20.0	95.0	94.3	98.8	24.2	2.30	6.90
<0.40 mm	3.0	80.0	5.0	5.7	1.2	75.8	97.7	93.1
Global size classification	Coarse	Fine-coarse	Coarse	Coarse	Coarse	Fine-coarse	Fine	Fine

Numbers 1 to 3 correspond to different commercial sources. MCP1: Global Feed, Spain; MCP2: Yara, Finland; MCP3: Aliphos, Belgium; DCP1: Global Feed, Spain; DCP2: Phosphea, France; DCP3: Aliphos, Belgium.

**Table 2:** Ingredients of the basal diet used in Experiments 1 and 2.

Ingredients	g/kg
Corn grain	375
Corn starch	268
Potato protein	162
Soybean meal 44% crude protein	83
Oat hulls	54
Soy oil	20
DL-methionine	2.7
L-arginine	2.5
Sodium bicarbonate	5.0
Sodium chloride	1.7
Vitamin-mineral premix ^1^	6.0
Titanium dioxide	5.0
Limestone	3.4
Diatomaceous earth (Celite)	12.2

^1^ Composition per kg of premix: calcium 162.47 g/kg; E5 manganese (manganous oxide) 16,000.00 mg/kg; E6 zinc (zinc oxide) 103,60.00 mg/kg; E4 copper (cupric sulphate pentahydrate) 1250.00 mg/kg; E2 iodine (potassium iodide) 300.00 mg/kg; E8 selenium (sodium selenite) 25.00 mg/kg; E1 iron (ferrous carbonate) 4000.00 mg/kg; E672 vitamin A 1,200,000.00 UI/kg; E671 vitamin D3 240,000.00 UI/kg; 3a700 vitamin E/all-rac-alpha-tocopherol acetate 1200.00 UI/kg; vitamin K 320.00 mg/kg; vitamin B2 880.00 mg/kg; 3a831 vitamin B6/pyridoxine hydrochloride 300.00 mg/kg; vitamin B12 2000.00 mcg/kg; 3a315 niacinamide 4000.00 mg/kg; calcium pantothenate 1345.00 mg/kg; pantothenic acid 1237.40 mg/kg; 3a316 folic acid 80.00 mg/kg; 3a890 choline chloride 47,500.00 mg/kg; betaine 10,830.00 mg/kg; E562 sepiolite 319.15 g/kg; E320 butylated hydroxyanisole (BHA) 100.00 mg/kg; E321 butylhydroxytoluene (BHT) 1100.00 mg/kg; E324 ethoxyquin 160.00 mg/kg; dry matter 960.55 g/kg.

**Table 3:** Calculated and analyzed nutrient composition of experimental diets (g/kg, as-fed basis).

		Experiment 1				Experiment 2					
	Basal Diet	MCP1 Level 1	MCP1 Level 2	DCP1 Level 1	DCP1 Level 2	MCP1 Level 2	MCP2 Level 2	MCP3 Level 2	DCP1 Level 2	DCP2 Level 2	DCP3 Level 2
Calculated composition											
Metabolizable energy (Kcal/kg)	3134	3134	3134	3134	3134	3134	3134	3134	3134	3134	3134
Crude protein	190	190	190	190	190	190	190	190	190	190	190
Total phosphorus (P)	2.25	3.01	3.76	3.01	3.76	3.76	3.76	3.76	3.76	3.75	3.75
Available P	0.69	1.32	1.95	1.23	1.77	1.94	1.95	1.95	1.77	1.77	1.77
Total calcium (Ca)	3.00	4.02	5.03	4.03	5.05	5.03	5.03	5.05	5.05	5.02	5.04
Ca: P	1.33	1.34	1.34	1.34	1.35	1.34	1.34	1.34	1.34	1.34	1.34
Analyzed composition											
Dry matter	897	894	895	895	894	894	894	894	894	894	895
Total P	2.25	2.90	3.45	2.95	3.70	4.10	3.75	3.60	3.60	3.70	3.80
Total Ca	3.00	4.00	4.70	3.95	4.80	5.10	4.85	4.65	4.75	4.95	5.05
Ca:P	1.33	1.38	1.36	1.34	1.30	1.24	1.29	1.29	1.32	1.34	1.33

MCP, monocalcium phosphate and DCP, dicalcium phosphate; 1 to 3 correspond to different commercial sources described in Table 1. Level 1: 0.75 g of added P from the test source/kg; Level 2: 1.50 g of added P from the test source/kg.

**Table 4:** Effect of phosphate level and source on main performance and bone mineralization traits of birds fed a low-phosphorus (P) basal diet and graded levels of dietary P from test ingredients from 15 to 25 days in Experiment 1 (*n* = 8 cages of 2 birds per treatment).

	Basal Diet	MCP1 Level 1	MCP1 Level 2	DCP1 Level 1	DCP1 Level 2	SEM ^1^	*p*-Value
Performance traits:							
Body weight at 15 days, g	494.7	484.0	486.7	487.9	487.4	11.90	0.971
Body weight at 25 days, g	782.0	856.9	788.9	817.1	814.3	26.43	0.239
Average daily feed intake, g/day	50.2	58.3	50.8	54.8	52.7	2.80	0.190
Average daily gain, g/day	29.4	36.9	30.1	32.9	32.6	2.64	0.240
Feed conversion ratio	1.74	1.61	1.75	1.68	1.63	0.059	0.266
Bone mineralization traits:							
Tibia weight, g	1.49 ^b^	1.66 ^ab^	1.73 ^a^	1.65 ^ab^	1.74 ^a^	0.048	0.004
Tibia ash, %	36.5 ^c^	39.8 ^b^	42.2 ^a^	39.0 ^b^	42.0 ^a^	0.56	<0.001
P in ash, %	16.5	16.8	16.8	16.8	16.8	0.11	0.1026
Ca in ash, %	34.9	35.1	35.3	35.7	35.3	0.23	0.126
P, mg in tibia	87.3 ^b^	110.5 ^a^	122.8 ^a^	108.4 ^a^	122.2 ^a^	3.97	<0.001
Ca, mg in tibia	185.1 ^b^	231.9 ^a^	256.8 ^a^	230.4 ^a^	257.4 ^a^	8.21	<0.001

Sources: MCP, monocalcium phosphate and DCP, dicalcium phosphate; the large number (1) corresponds to the commercial source described in Table 1; subscripts represent the level of total P added to each experimental diet (g/Kg). Level 1: 0.75 g of added P from the test sources/kg; Level 2: 1.5 g of added P from the test source/kg. Basal diet (BS) diet without added P; MCP1 Level 1: BS + 0.75 g of added P from MCP1/kg; MCP1 Level 2: BS + 1.50 g of added P from MCP1/kg; DCP1 Level 1: BS + 0.75 g of added P from DCP1/kg; DCP1 Level 2: BS + 1.50 g of added P from DCP1/kg. ^1^ SEM: standard error of the mean. ^a–c^ Values within a row with no superscript in common are statistically different (*p* ≤ 0.05).

**Table 5:** Digestibility coefficients of dry matter (DM), phosphorus (P) and calcium (Ca) of birds fed a low-P basal diet and graded levels of dietary P from test ingredients from 21 to 25 days in Experiment 1 (*n* = 8 cages of 2 birds per treatment).

	Basal Diet	MCP1 Level 1	MCP1 Level 2	DCP1 Level 1	DCP1 Level 2	SEM ^1^	*p*-Value
Dry matter digestibility (%)							
ATTD-tc of DM	78.1 ^b^	79.9 ^a^	80.2 ^a^	78.6 ^a^	79.5 ^a^	0.55	0.038
ATTD-m of DM	80.1 ^ab^	79.8 ^abc^	79.5 ^bc^	79.1 ^c^	80.7 ^a^	0.26	0.001
pc-D of DM	80.9	79.1	78.7	79.6	80.3	1.03	0.529
Phosphorous digestibility (%)							
ATTD-tc of P	46.5 ^b^	57.6 ^a^	61.6 ^a^	58.2 ^a^	60.2 ^a^	2.56	<0.001
ATTD-m of P	50.1 ^b^	57.3 ^ab^	60.2 ^a^	59.0 ^ab^	62.3 ^a^	2.08	0.012
pc-D of P	45.9 ^b^	58.8 ^a^	62.8 ^a^	59.4 ^a^	62.6 ^a^	2.83	<0.001

Sources: MCP, monocalcium phosphate and DCP, dicalcium phosphate; the large number (1) corresponds to the commercial source described in Table 1; subscripts represent the level of total P added to each experimental diet (g/Kg). Level 1: 0.75 g of added P from the test sources/kg; Level 2: 1.5 g of added P from the test source/kg. Basal diet (BS) diet without added P; MCP1 Level 1: BS + 0.75 g of added P from MCP1/kg; MCP1 Level 2: BS + 1.50 g of added P from MCP1/kg; DCP1 Level 1: BS + 0.75 g of added P from DCP1/kg; DCP1 Level 2: BS + 1.50 g of added P from DCP1/kg. Digestibility coefficients: ATTD-tc, apparent total tract digestibility using total collection method; ATTD-m, ATTD using marker method; pc-D, prececal digestibility. ^1^ SEM: standard error of the mean. ^a–c^ Values within a row with no superscript in common are statistically different (*p* ≤ 0.05).

**Table 6:** Slope comparison of linear regression of phosphorous (P) digestibility as a function of the mineral source (MCP or DCP) and calculation method (ATTD-tc, ATTD-m or pc-D) in Experiment 1.

Phosphorus Digestibility Method	Regression Equation	SE ^1^ of the Slope	r^2^	P Digestibility Coefficient (%)
Monocalcium phosphate, MCP				
ATTDP-tc	1.188 + 0.835 × Padded ^2^	0.054	0.926	83.48
ATTDP-m	1.266 + 0.752 × Padded	0.057	0.901	75.21
pc-DP	1.183 + 0.874 × Padded	0.070	0.886	87.42
*p*-value slope comparison				0.188
Dicalcium phosphate, DCP				
ATTDP-tc	1.208 + 0.808 × Padded	0.070	0.875	80.76
ATTDP-m	1.274 + 0.805 × Padded	0.067	0.899	80.45
pc-DP	1.197 + 0.866 × Padded	0.082	0.862	86.62
*p*-value slope comparison	-	-	-	0.560

Sources: MCP, monocalcium phosphate and DCP, dicalcium phosphate; digestibility method: ATTD-tc, apparent total tract digestibility using total collection method; ATTD-m, ATTD using marker method; pc-D, prececal digestibility. ^1^ SE: standard error. ^2^ Padded: phosphorus added from the test source (g/kg of diet).

**Table 7:** Effect of commercial phosphate source on main performance and bone mineralization traits of birds fed a low-phosphorus (P) basal diet and graded levels of dietary P from test ingredients from 15 to 25 days in Experiment 2 (*n* = 8 cages of 2 birds per treatment).

Parameters	MCP1 Level 2	MCP2 Level 2	MCP3 Level 2	SEM ^1^	*p*-Value	DCP1 Level 2	DCP2 Level 2	DCP3 Level 2	SEM	*p*-Value
Performance traits:										
Body weight at 15 days, g	487.7	486.4	486.4	9.89	0.994	486.5	488.6	490.9	10.51	0.957
Body weight at 25 days, g	834.6	845.7	802.6	26.08	0.450	808.1	839.9	824.0	26.75	0.672
Average daily feed intake, g/day	55.4	59.5	52.7	2.86	0.267	52.7	54.2	55.3	2.79	0.809
Average daily gain, g/day	34.8	37.8	31.6	2.56	0.261	32.1	35.2	33.7	2.67	0.672
Feed conversion ratio	1.64	1.60	1.70	0.054	0.427	1.67	1.59	1.78	0.078	0.240
Bone mineralization traits:										
Tibia weight, g	1.81	1.72	1.74	0.061	0.494	1.69	1.74	1.70	0.036	0.552
Tibia weight, %BW	0.22	0.20	0.22	0.004	0.065	0.22	0.22	0.21	0.006	0.834
Tibia ash, %	42.2	41.9	41.9	0.40	0.811	41.9	41.3	41.6	0.370	0.596
P in ash, %	16.9	16.8	16.9	0.08	0.537	16.9	16.9	16.8	0.122	0.765
Ca in ash, %	35.1	34.9	35.4	0.22	0.356	35.5	35.6	35.2	0.287	0.483
P, mg	128.8	120.9	122.9	3.84	0.302	120.3	121.6	118.2	2.44	0.630
Ca, mg	267.9	251.7	256.8	7.46	0.275	252.9	255.9	247.5	5.58	0.566

Sources: MCP, monocalcium phosphate and DCP, dicalcium phosphate; the large numbers (1, 2 and 3) correspond to the commercial source described in Table 1; subscripts represent the level of total P added to each experimental diet (g/Kg). Level 2: 1.5 g of added P from the test source/kg. MCP1 Level 2: basal diet (BS) + 1.50 g of added P from MCP1/kg; MCP2 Level 2: BS + 1.50 g of added P from MCP2/kg; MCP3 Level 2: BS + 1.50 g of added P from MCP3/kg; DCP1 Level 2: BS + 1.50 g of added P from DCP1/kg; DCP2 Level 2: BS + 1.50 g of added P from DCP2/kg; DCP3 Level 2: BS + 1.50 g of added P from DCP3/kg. Basal diet is the same as in Experiment 1. ^1^ SEM: Standard error of the mean.

**Table 8:** Digestibility coefficients of dry matter (DM) and phosphorus (P) of birds fed dietary P from different commercial sources from 21 to 25 days in Experiment 2 (*n* = 8 cages of 2 birds per treatment).

Digestibility Coefficients	MCP1 Level 2	MCP2 Level 2	MCP3 Level 2	SEM ^1^	*p*-Value	DCP1 Level 2	DCP2 Level 2	DCP3 Level 2	SEM	*p*-Value
ATTD-tc of DM, %	79.6	78.5	77.9	1.03	0.442	78.8	79.4	79.4	0.42	0.525
ATTD-tc of P, %	59.8	56.3	54.2	2.48	0.249	60.0	58.2	56.5	1.06	0.092

Sources: MCP, monocalcium phosphate and DCP, dicalcium phosphate; the large numbers (1, 2 and 3) correspond to the different commercial sources described in Table 1; subscripts represent the level of total P added to each experimental diet (g/Kg). Level 2: 1.5 g of added P from the test source/kg. MCP1 Level 2: basal diet (BS) + 1.50 g of added P from MCP1/kg; MCP2 Level 2: BS + 1.50 g of added P from MCP2/kg; MCP3 Level 2: BS + 1.50 g of added P from MCP3/kg; DCP1 Level 2: BS + 1.50 g of added P from DCP1/kg; DCP2 Level 2: BS + 1.50 g of added P from DCP2/kg; DCP3 Level 2: BS + 1.50 g of added P from DCP3/kg. Basal diet is the same as in Experiment 1. Digestibility coefficients: ATTD-tc, apparent total tract digestibility using the total collection method. ^1^ SEM: standard error of the mean.

**Table 9:** Linear relationship between digestible phosphorus (P) content (g/kg) using the total collection method (ATTD-tc) and dietary P concentration (g/kg dry matter, DM) of test ingredients in Experiment 2.

	Diets			
Test Ingredient	Regression Equation	SE ^1^ of the Slope	r^2^	P Digestibility Coefficient (%)
Monocalcium phosphate				
MCP1 Level2	1.167 + 0.796 × Padded ^2^	0.024	0.997	79.58
MCP2 Level2	1.167 + 0.702 × Padded	0.032	0.994	70.15
MCP3 Level2	1.167 + 0.656 × Padded	0.068	0.971	65.58
*p*-value slope comparison				0.151
Dicalcium phosphate				
DCP1 Level2	1.167 + 0.801 × Padded	0.026	0.996	80.06
DCP2 Level2	1.167 + 0.774 × Padded	0.034	0.994	77.37
DCP3 Level2	1.167 + 0.714 × Padded	0.030	0.995	71.44
*p*-value slope comparison	-	-	-	0.110

Sources: MCP, monocalcium phosphate and DCP, dicalcium phosphate; the large numbers (1, 2 and 3) correspond to the different commercial sources described in Table 1; subscripts represent the level of total P added to each experimental diet (g/Kg). Level 2: 1.5 g of added P from the test source/kg. MCP1 Level 2: basal diet (BS) + 1.50 g of added P from MCP1/kg; MCP2 Level 2: BS + 1.50 g of added P from MCP2/kg; MCP3 Level 2: BS + 1.50 g of added P from MCP3/kg; DCP1 Level 2: BS + 1.50 g of added P from DCP1/kg; DCP2 Level 2: BS + 1.50 g of added P from DCP2/kg; DCP3 Level 2: BS + 1.50 g of added P from DCP3/kg. Basal diet is the same as in Experiment 1. ^1^ SE: standard error. ^2^ Padded: phosphorus added from the test source (g/kg of diet).

**Table 10:** Scanning electron X-ray analyses of monocalcium phosphate (MCP) samples.

Parameters	MCP1 Level 2	MCP2 Level 2	MCP3 Level 2	SEM ^1^	*p*-Value
Sodium	1.60 ^a^	0.00 ^b^	0.00 ^b^	0.233	0.0004
Magnesium	1.29 ^b^	6.02 ^a^	7.12 ^a^	0.781	0.0004
Aluminium	0.45 ^b^	2.00 ^a^	0.25 ^b^	0.230	0.0003
Silicon	0.00 ^b^	0.00 ^b^	0.51 ^a^	0.052	<0.0001
Sulphur	0.08	0.11	0.19	0.075	0.543
Potassium	0.17 ^b^	1.79 ^a^	0.12 ^b^	0.112	<0.0001
Iron	0.09 ^b^	2.70 ^a^	0.21 ^b^	0.173	<0.0001

The large numbers (1, 2 and 3) correspond to the commercial source described in Table 1; subscripts represent the level of total P added to each experimental diet (g/Kg). Level 2: 1.5 g of added P from the test source/kg. ^1^ SEM: standard error of the mean.

**Table 11:** Scanning electron X-ray analyses of dicalcium phosphate (DCP) samples.

Parameters	DCP1 Level 2	DCP2 Level 2	DCP3 Level 2	SEM ^1^	*p*-Value
Sodium	0.08	0.00	0.00	0.034	0.197
Magnesium	1.15 ^a^	0.15^c^	0.74 ^b^	0.113	<0.0001
Aluminium	0.30 ^a^	0.16 ^b^	0.15 ^b^	0.034	0.013
Silicon	0.01 ^b^	0.20 ^a^	0.15 ^ab^	0.048	0.033
Sulphur	2.06 ^ab^	0.52 ^b^	4.02 ^a^	0.989	0.028
Chlorine	0.00 ^b^	0.62 ^a^	0.00 ^b^	0.035	<0.0001
Potassium	0.11 ^a^	0.00 ^b^	0.00 ^b^	0.025	0.010
Iron	0.08	0.03	0.00	0.043	0.347
Copper	0.00	0.12	0.00	0.010	0.489

The large numbers (1, 2 and 3) correspond to the commercial source described in Table 1; subscripts represent the level of total P added to each experimental diet (g/Kg). Level 2: 1.5 g of added P from the test source/kg. ^1^ SEM: standard error of the mean.

## Data Availability

Not applicable.
